# Biodistribution and Dosimetry Evaluation for a Novel Tau Tracer [^18^F]-S16 in Healthy Volunteers and Its Application in Assessment of Tau Pathology in Alzheimer’s Disease

**DOI:** 10.3389/fbioe.2021.812818

**Published:** 2022-02-10

**Authors:** Ying Wang, Li Cai, Kaixiang Zhou, Mengchao Cui, Shaobo Yao

**Affiliations:** ^1^ Department of PET/CT Diagnostic, Tianjin Medical University General Hospital, Tianjin, China; ^2^ Key Laboratory of Radiopharmaceuticals, Ministry of Education, Beijing Normal University, Beijing, China; ^3^ Department of Nuclear Medicine, Fujian Provincial Key Laboratory of Precision Medicine for Cancer, The First Affiliated Hospital of Fujian Medical University, Fuzhou, China

**Keywords:** radiation dosimetry, Alzheimer’s disease, tau, [^18^F]-S16, PET/CT

## Abstract

**Background:** The goal of this study was to report a fully automated radiosynthetic procedure of a novel tau tracer [^18^F]-S16 and its safety, biodistribution, and dosimetry in healthy volunteers as well as the potential utility of [^18^F]-S16 positron emission tomography (PET) in Alzheimer’s disease (AD).

**Methods:** The automated radiosynthesis of [^18^F]-S16 was performed on a GE Tracerlab FX2 N module. For the biodistribution and dosimetry study, healthy volunteers underwent a series of PET scans acquired at 10, 60, 120, and 240 min post-injection. The biodistribution and safety were assessed. For the AD study, both AD and healthy controls (HCs) underwent dynamic [^18^F]-S16 and static [^18^F]-FDG PET imaging. [^18^F]-S16 binding was assessed quantitatively using standardized uptake value ratios (SUVRs) measured at different regions of interest (ROIs). [^18^F]-S16 SUVRs were compared between the AD patients and HCs using the Mann–Whitney *U*-test. In AD patients with all cortical ROIs, Spearman rank-correlation analysis was used to calculate the voxel-wise correlations between [^18^F]-S16 and [^18^F]-FDG.

**Results:** The automated radiosynthesis of [^18^F]-S16 was finished within 45 min, with a radiochemical yield of 30 ± 5% (*n* = 8, non-decay-corrected). The radiochemical purity was greater than 98%, and the specific activity was calculated to be 1,047 ± 450 GBq/μmol (*n* = 5), and [^18^F]-S16 was stable *in vitro*. In the healthy volunteer study, no adverse effect was observed within 24 h post-injection, and no defluorination was observed *in vivo*. The radiotracer could pass through the blood–brain barrier easily and was rapidly cleared from the circulation and excreted through the hepatic system. The whole-body mean effective dose was 15.3 ± 0.3 μSv/MBq. In AD patients, [^18^F]-S16 accumulation was identified as involving the parietal, temporal, precuneus, posterior cingulate, and frontal lobes. No specific [^18^F]-S16 cerebral uptake was identified in HCs. The SUVR of AD patients was significantly higher than that of HCs. No specific binding uptake was found in the choroid plexus, venous sinus, and white matter. A significant correlation was found between [^18^F]-S16 binding and hypometabolism across neocortical regions.

**Conclusion:** [^18^F]-S16 could be synthesized automatically, and it showed favorable biodistribution and safety in humans. [^18^F]-S16 PET indicated a high image quality for imaging tau deposition in AD and distinguishing AD from HCs.

## Introduction

Alzheimer’s disease (AD) is a progressive, neurodegenerative condition that results in both cognitive and functional decline. The enormous human and socioeconomic burden associated with AD has motivated an intense international research effort directed toward earlier and more accurate diagnosis and the development of disease-modifying treatments. Amyloid-β (Aβ) plaques, together with tau neurofibrillary tangles (NFTs), are the neuropathological hallmarks of AD (Dani et al., 2018). It has been proposed to classify AD according to the biomarkers for amyloid, tau, and neuronal injury by the A/T/N scheme (Jack et al., 2016).The correlations among amyloid accumulation, neurodegeneration, and clinical decline are not straightforward. Conversely, the distribution and burden of NFTs show a closer association with neurodegeneration and clinical status (Spires-Jones and Hyman, 2014).

Tau is a complex protein with multiple isoforms and post-translational modifications. Tracers may bind to specific or multiple isoforms. As tau is an intracellular protein, radioligands must cross the cell membrane of neurons. In addition, Aβ and tau both manifest a β-sheet structure which binds planar polyaromatic ligands. A selective ligand needs to have at least 10-fold higher binding affinity for tau compared with Aβ (Dani et al., 2016). In the recent 10 years, a number of tau ligands for *in vivo* positron emission tomography (PET) imaging have been developed and evaluated in both preclinical and clinical studies. Tau-PET imaging has opened a unique window to expand our insight into the pathology of AD and other tau-related neurodegenerative diseases. Representative tau-PET ligands, including the first-generation tracers—THK tracers, T807/T808, and [^11^C]-PBB3—and the second-generation tracers—[^18^F]-MK-6240, [^18^F]APN-1607, and [^18^F]PI-2620—can reflect the timing and distribution of tau in the early phases of neurodegenerative diseases (Fodero-Tavoletti et al., 2011; Chien et al., 2014; Marquié et al., 2015; Walji et al., 2016; Marquié et al., 2017).

However, almost all of the first-generation tau tracers mentioned above presented the same drawback, various degrees of what has been called “off-target” binding. This means the retention of radiotracers in brain areas without tau deposition, such as the basal ganglia, the choroid plexus, or the thalamus (Marquié et al., 2015; Marquié et al., 2017). In addition, the THK tracers—including [^18^F]-THK523, [^18^F]-THK5317, and [^18^F]-THK5351—displayed some white matter retention which hindered an accurate visual interpretation of signals (Betthauser et al., 2017; Hsiao et al., 2017). [^18^F]-T808 showed high binding affinity and good selectivity for tau over Aβ, with rapid uptake and washout in transgenic mice. However, high defluorination *in vivo* hindered its further development and application in clinical studies (Chien et al., 2014). The drawbacks of [^11^C]-PBB3 include its short half-life requiring on-site synthesis and the formation of *cis*-trans isomers under the light during radiosynthesis (Hashimoto et al., 2014; Kimura et al., 2015; Ni et al., 2018). Initial human studies of some second-generation tracers such as [^18^F]-MK6240 have shown no “off-target” binding so far. However, further detailed clinical trials were still needed (Betthauser et al., 2018).

We had developed a novel tau PET radiotracer [^18^F]-S16 ([^18^F]-(S)-1-(4-(6-(dimethylamino)quinoxalin-2-yl)phenoxy)-3-fluoropropan-2-ol) with a 2-phenylquinoxaline scaffold. [^18^F]-S16 displayed high affinity and selectivity for tangles over Aβ as well as sufficient blood–brain barrier penetration and rapid brain washout in normal mice (Zhou et al., 2021). The goal of this study was to report a fully automated radiosynthetic procedure of [^18^F]-S16 and to evaluate its safety, biodistribution, and dosimetry in healthy volunteers. Moreover, we aimed to identify the [^18^F]-S16 binding patterns in AD patients relative to sex- and age-matched healthy controls (HCs) and the relationship between cerebral tau pathology and hypometabolism patterns with dual [^18^F]-S16 (T) and [^18^F]-FDG (N). The flow chart of the study design is shown in Supplementary Figure S1.

## Materials and Methods

### General

All chemicals obtained commercially were of analytical grade (Sigma-Aldrich, United States) and used without further purification unless otherwise stated. The tosylate precursor and [^19^F]-labeled reference ligand were supplied by Prof. Cui. Sep-Pak light QMA and Sep-Pak plus (light) C18 cartridges were obtained from Waters (Milford, United States). The Sep-Pak light QMA cartridges were pre-conditioned with 8.4% NaHCO_3_ (8 ml) and water (10 ml) before use. The Sep-Pak C18 plus cartridges were preconditioned with ethanol (10 ml) and water (10 ml) in advance. Fetal bovine serum was purchased from HyClone (Thermo Scientific, United States) and stored under −20°C before use.

### Automated Radiosynthesis of [^18^F]-S16

[^18^F]-S16 was prepared by a one-pot two-step reaction described in Supplementary Figure S2. The automated radiosynthesis of [^18^F]-S16 was performed on the GE Tracerlab FX2 N synthesis unit (GE Healthcare, United States). No-carrier-added [^18^F]-fluoride was produced through the nuclear reaction ^18^O (p, n) ^18^F by irradiation of more than 95% [^18^O] enriched water target with 10 MeV proton beam on the GE MINItrace cyclotron. Millexs-GV 0.22-μm-filter units were purchased from Merck Milipore Ltd. The analysis radio-HPLC system was equipped with a gamma ray radiodetector and a UV detector [Varian system: Waters Symmetry C18 column (5 μm, 4.6 × 250 mm); mobile phase: 1 ml/min with an eluent of CH_3_CN/H_2_O 50/50]. Radioactivity was measured by CRC-15PET Radioisotope Dose Calibrator (Capintec. Inc., United States).

Before production, vial 1 was filled with a mixture of Kryptofix 222 (K_222_, 13 mg), potassium carbonate (K_2_CO_3_, 1.1 mg), CH_3_CN (0.3 ml), and H_2_O (0.3 ml); vial 2 was filled with anhydrous CH_3_CN (1 ml); vial 3 was filled with compound **1** (3 mg) dissolved in anhydrous CH_3_CN (1.5 ml); vial 4 was filled with 0.5 M HCl (0.5 ml); vial 5 was filled with 0.5 M NaOH (0.5 ml); vial 6 was filled with 2.5 ml CH_3_CN/H_2_O (60/40) mixture; vial 12 was filled with saline (10 ml); vial 13 was filled with ethanol (1 ml); vial 14 was filled with H_2_O (10 ml); and the round bubble vessel at the bottom-right was filled with 50 ml H_2_O.

After [^18^F]-fluoride (800–1,000 mCi) was delivered from the cyclotron and trapped on QMA, the excess ^18^H_2_O was removed. The trapped [^18^F] activity was eluted to the reactor 1 with K_222_ elution (0.6 ml, vial 1). The reaction mixture was evaporated under a stream of nitrogen (80 ml/min) at 95°C under reduced pressure. The residue was azeotropically dried with CH_3_CN (1 ml, vial 2) at 95°C again. Then, a solution of compound 1 (3 mg) in anhydrous CH_3_CN (1.5 ml, vial 3) was added, and fluorination was carried out at 100°C for 5 min. The reaction mixture was bubbled with N_2_ to dryness. HCl solution (0.5 ml, vial 4) was added to the reaction vessel, and this was heated at 100°C for 3 min. After adding NaOH solution (0.5 ml, vial 5) and a mixture of CH_3_CN and H_2_O (2.5 ml, vial 6), the mixture was loaded to the semi-prep HPLC system (Syknm S1122 Solvent Delivery System) equipped with a gamma ray radiodetector and a UV detector [Machery-Nagel Nucleosil 100-7 C18 column (16 × 250 mm); mobile phase: 16 ml/min with an eluent of CH_3_CN/H_2_O, 60/40]. After the collection of product gradient, the elution was diluted with H_2_O (50 ml) in the bubble vessel and transferred to the C18 cartridge. The C18 cartridge was washed with H_2_O (10 ml, vial 14) to remove high-polarity impurities and dried with helium. The radioactivity was eluted by ethanol (1 ml, vial 13) and diluted by saline (10 ml, vial 12). [^18^F]-S16 saline was filtered through a sterile Millipore GV filter (0.22 μm, 25 mm) directly into a sterile product vial (20-ml size).

Chemical identification of the purified product was carried out by HPLC co-injection with the non-radioactive reference [^19^F]-S16, using UV detector at 254 nm UV and a gamma ray radiodetector. The analysis radio-HPLC condition was the same as mentioned above.

For *in vitro* assays, 0.1-ml samples of [^18^F]-S16 (1.85 MBq, 50 μCi) were dissolved in sterile saline and incubated with 0.2 ml of fetal bovine serum (FBS) at 37°C with gentle shaking. An aliquot of the injection saline and the serum sample was analyzed by radio-HPLC to determine the percentage of intact [^18^F]-S16 at 120 min.

### Participants and Methods

This clinical study was approved by the Institute Review Board of Tianjin Medical University General Hospital (IRB2018-072-01). Participant recruitment and the clinical trial were conducted from August 10, 2018 to August 10, 2020. The trial was registered on Clinicaltrial.gov with the identification number NCT03620552.

In the pilot study, four healthy volunteers with the age of 49.25 ± 11.52 years [three men and one woman, Mini-Mental State Examination (MMSE) 30] were enrolled after giving their written informed consent. These subjects were deemed to be in good health based on their clinical history, physical examination, standard blood and urine tests, and electrocardiogram. The subjects were contacted by telephone approximately 24 h after [^18^F]-S16 administration to monitor adverse events.

In the AD diagnosis study, 15 probable AD patients and six age- and sex-matched HCs were recruited from research cohorts of the Department of Neurology. They all received a standard clinical evaluation, including a comprehensive neurological history and physical and neurological examinations (brain MRI and neuropsychological assessment with a battery of tests). All the subjects were Han Chinese. The clinical diagnosis was determined by consensus of a multidisciplinary team. The diagnosis of probable AD was based on the NINCDS/ADRDA and DSM-IV criteria. At screening, the Clinical Dementia Rating (CDR) score was at least 0.5, and the MMSE score was no more than 28. They all had positive [^11^C]-Pittsburgh compound B ([^11^C]-PiB) PET scan (assessing *in vivo* amyloid pathology) under the published procedures (Wang et al., 2019). The inclusion criteria for HCs were no history of major psychiatric or neurological illnesses, no head injury, and no family history of AD. The MMSE score was more than 28, and the CDR score was 0. The HCs had negative [^11^C]-PiB PET scans. All participants (or their legal representatives) provided written informed consent to participate in the study. The demographics and clinical data are summarized in Supplementary Table S1.

### [^18^F]-S16 and [^18^F]-FDG PET/CT

In the pilot study, four healthy volunteers underwent a total of four sequential whole-body [^18^F]-S16 PET/CT scans from the base of the skull to the proximal thigh using Discovery PET/CT 710 scanner (GE Healthcare, Milwaukee, WI, United States). The [^18^F]-S16 radiotracer was injected through a venous line into the arm of healthy volunteers, with a mean administered activity of 444 ± 37 MBq. The radiotracers were administered to the subjects by a rapid bolus injection (5 ml over 15 s). The low-dose CT scan of each subject for location and attenuation correction was obtained from the base of the skull to the proximate thigh (CT settings: 120 keV; ^30m^As; pitch, 0.984; slice thickness, 3.75 mm; rotation time, 0.8 s). The scanning protocol included 2-min emission scans for four cycles at 10, 60, 120, and 240 min post-injection. Each cycle consisted of eight bed positions. Thus, the whole-body scanning time was 16 min per cycle. A 10-min static brain scan was also performed, following the second whole-body scan, at approximately 76 min after injection. All data were decay-corrected to the starting time of each individual scan. All PET images were corrected for photon attenuation, dead time, random events, and scatter. Images were reconstructed in the three-dimensional mode using an OSEM + PSF + TOF reconstruction technique (ordered-subset expectation maximization with 24 subsets and two iterations). The image matrix was 256 × 256 (pixel size, 2.73 mm), and the slice thickness was 3.75 mm.

In the AD diagnosis study, each subject completed the [^18^F]-S16 and [^18^F]-FDG PET scans within 1 week. The participants, who were given an intravenous injection of [^18^F]-S16 (383.6 ± 30.6 MBq), participated in an acquisition scheme: 70–100 min (5 frames: 6 × 300 s). A few days later, after having fasted for at least 6 h, the participants were injected with about 259 MBq [^18^F]-FDG, and 10-min static PET scans were acquired at 40 min post-injection. Each frame produced 47 slices of 3.75-mm thickness, which covered the whole brain. The images were reconstructed to 256 × 256 matrix.

### Safety, Biodistribution, and Radiation Dosimetry

Safety and tolerability were assessed, including blood and urine samples for laboratory tests, electrocardiograms, and physical and clinical examinations. Adverse events were assessed by telephone approximately 24 h after the examination. Whole-organ volumes of interest were drawn manually over the source organs, including spleen, liver, kidneys, pituitary glands, vertebral bodies L1–L5, and urinary bladder, at each time point. The non-decay-corrected activities at different time points were documented as the percentage of injected dosage and fitted with mono-exponential curves. The area under the time–activity curve between time 0 and the first time point was calculated assuming a linear increase from 0 to the first measured activity. The area under the time–activity curve after the first time point was calculated by trapezoidal integration from the first time point to the last time point and extrapolation from the last data point using the fitted mono-exponential function. For bone marrow, the residence time was derived by an image-based integration of L1–L5 vertebra, assuming L1–L5 to be with 12.3% of the whole-body bone marrow. The urinary bladder residence time was determined by using the voiding bladder model implemented in OLINDA/EXM software, with 2 h set as the bladder voiding interval. The residence time of the rest of the body was calculated as the maximum possible residence time (based on physical decay only) minus the sum of the residence time of all source organs. The absorbed dose of target organs and the whole-body effective dose were measured with OLINDA/EXM software by adult male models.

### Region of Interest Analyses for [^18^F]-S16

In the pilot study, three experienced nuclear medicine physicians read all of the images through consensus reading. The same nuclear medicine physicians examined and measured the semiquantitative values for the final analysis. GE MMWP workstation was used for postprocessing. The physiologic uptake of the following organs was evaluated at all time points: brain, salivary gland, heart, liver, spleen, bone marrow, kidneys, urinary bladder, and small intestine. Regions of interest were drawn over these organs to exclude focal lesions, and the maximum standardized uptake value (SUV_max_) and mean standardized uptake value (SUV_mean_) normalized to the body weight of the patients were recorded.

In the AD diagnosis study, images were analyzed in the software package PMOD (version 3.7, PMOD Technologies Ltd., Zurich, Switzerland). Dynamic [^18^F]-S16 images were co-registered to the standard Montreal Neurologic Institute (MNI) space by applying the 70–100-min transformation (brain normalization settings: nonlinear warping, 8-mm input smoothing, 16 iterations, frequency cutoff: 3, regularization: 1.0, no thresholding). All images were analyzed in MNI space. A total number of nine predefined regions of interest (ROIs), deriving from the Hammers atlas (Ossenkoppele et al., 2018), were delineated in the MNI space, including frontal (superior and middle gyri and orbitofrontal cortex), lateral temporal (superior, middle, and inferior gyri), parietal (inferior, superior, and supmarginal gyri), occipital (calcarine, cuneus, and lateral occipital cortex), posterior cingulate and precuneus, medial temporal [entorhinal cortex, hippocampus, and parahippocampus, medial temporal lobe (MTL)], lentiform (putamen and pallidum), and thalamus and white matter. The mean standardized uptake value ratios (SUVRs) of [^18^F]-S16 were calculated by using mean activity in the cerebellar gray matter as the reference region. These regions were selected as we aimed to assess the full spectrum of the Braak stages.

For [^18^F]-S16, non-displaceable binding potential (BP_ND_), a measure of specific binding, was determined for each Hammers atlas region of interest using a basis function implementation of the simplified reference tissue model operating upon the Hammers atlas and reference tissue ROI data. Dynamic 70–100 min [^18^F]-S16 data were analyzed using the non-invasive Logan graphical analysis (*k*_2_′ = 0.05 min^−1^, t* = 80 min), which were used to determine directly BP_ND_ using the cerebellar gray matter as the reference region.

### Region of Interest Analyses for [^18^F]-FDG PET

The mean SUVRs of [^18^F]-FDG were calculated for 10-min static data by using mean activity in the pons as the reference region. SUVs were calculated by normalizing the uptake values by the injected dose divided by the subject weight. Target-to-pons SUVRs were calculated for 10-min static data and compared with [^18^F]-S16 SUVRs.

### Statistical Analysis

The comparison of demographics between AD and HC groups was performed by one-way analysis of variance across groups and Fisher’s exact test. The [^18^F]-S16 SUVR data were compared between the AD and HC groups in different ROIs using the Mann–Whitney *U*-test. In AD patients with all cortical ROI regions, Spearman rank-correlation analysis was used to calculate the voxel-wise correlations between [^18^F]-S16 and [^18^F]-FDG PET. A significance level of *p* <0.05 was applied in all analyses. All statistical analyses were performed using SPSS (version 25.0, IBM, Armonk, New York, United States).

## Results

### Automated Radiosynthesis of [^18^F]-S16

[^18^F]-S16 was synthesized for 45 min with a high radiochemical yield of 30 ± 5% (*n* = 8, non-decay-corrected). The identity of [^18^F]-S16 was confirmed by a co-injection of [^19^F]-S16 as a standard (Supplementary Figures S3A,B). The radiochemical purity of [^18^F]-S16 injection was greater than 98%, and the specific activity was 1,047 ± 450 GBq/μmol (*n* = 5) (Supplementary Figure S4). [^18^F]-S16 showed high *in vitro* stability in FBS and saline, and more than 93 and 96% of [^18^F]-S16 remained intact within 120 min (longer times were tested) (Supplementary Figures S3C,D).

### Safety and Biodistribution

**FIGURE 1:**
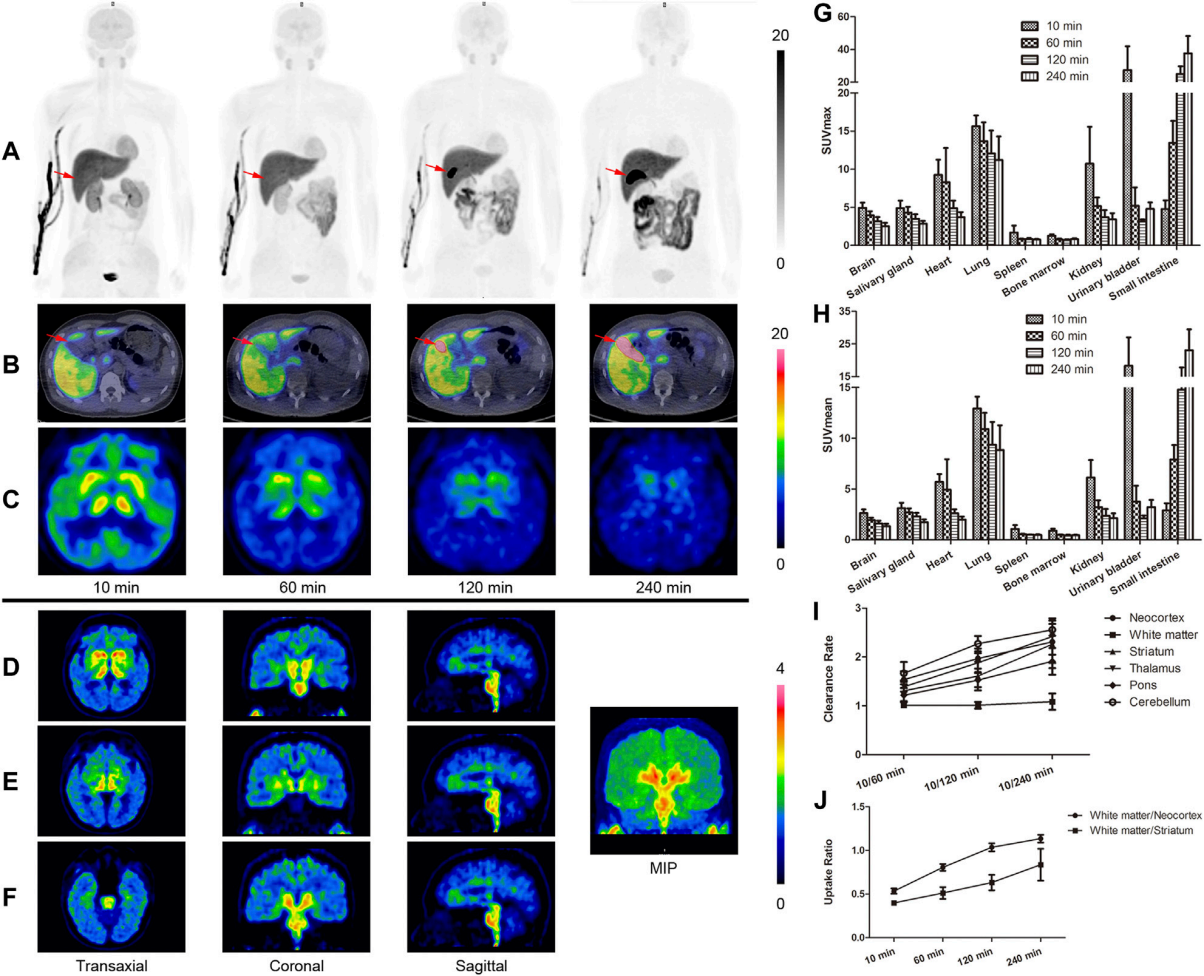
Whole-body positron emission tomography (PET) images of [^18^F]-S16 obtained in a healthy volunteer acquired over 8-bed positions of varying duration. The maximum intensity projection images of [^18^F]-S16 at 4 different time points after intravenous injection **(A)**. The time distribution pattern of [^18^F]-S16 in the gall bladder (**B**, red arrow) and in the brain **(C)**. The brain PET images obtained after the second whole-body scan, approximately 76 min post-injection, presented a transverse, coronal, and sagittal plane **(D**–**F)**. SUV_max_ **(G)** and SUV_mean_ **(H)** value of [^18^F]-S16 in the main organs of healthy volunteers (*n* = 4) at 10, 60, 120, and 240 min post-injection. Brain clearance rate (10/60, 10/120, and 10/240 min) **(I)** and white matter/necortex and white matter/striatum uptake ratio (10, 60, 120, and 240 min) **(H)** were summarized.

[^18^F]-S16 was found to be safe and well tolerated in all subjects. No adverse effects due to [^18^F]-S16 administration were observed in healthy volunteers up to 24 h after injection. The radiotracer was cleared mainly from the circulation and excreted into the gut through the hepatic–biliary system. The whole-body maximum intensity projection images of [^18^F]-S16 at four different time points (10–26, 60–76, 120–136, and 240–256 min) post-injection in a healthy volunteer are presented in Figure 1A. The probe was initially detected in the vascular compartment, then rapidly distributed through the extracellular space, and finally excreted through the liver, gall bladder (marked with red arrows), colon, and small intestine (Figures 1A,B). Consistent with the hydrophobic characteristic of [^18^F]-S16 (log *p*-value of 2.61 ± 0.05, reported in a previous paper), it was observed that [^18^F]-S16 accumulated significantly in the liver (hepatic system), not in the kidney (renal system). Moderate uptake was observed in the spleen, heart, brain, parotid gland, and salivary gland. Low radioactive uptake was observed in the lungs, pancreas, muscles, and bone marrow, which indicates high anti-defluorination stability *in vivo*. The quantitative biodistribution (SUV_max_ and SUV_mean_) in the main organs of healthy volunteers is summarized in Figures 1G,H. Static brain PET images are presented in Figures 1D-F, presented as transverse, coronal, and sagittal planes which were obtained following the second whole-body scanning at 76 min post-injection. No obvious radioactive uptake was found in the cerebral cortex, choroid, and sagittal sinus. Moderate radioactive uptake was observed mainly in the bilateral striatum, thalamus, and brainstem at 10 min post-injection, then decreased rapidly from 60 to 120 min, and was cleared from the blood pool at 240 min. The brain clearance rate, including neocortex, white matter, striatum, thalamus, pons, and cerebellum, was higher in 60, 120, and 240 min (Figure 1I). The uptake ratio of white matter/neocortex and white matter/striatum further increased during 10, 60, 120, and 240 min (Figure 1H).

### Radiation Dosimetry

The absorbed radiation doses of target organs are summarized in Table 1. The gallbladder wall received the highest radiation dose at 133.7 ± 60.9 μSv/MBq. The effective dose was 15.3 ± 0.3 μSv/MBq. The radioactive uptake of the hematopoietic system and genital system was low, which also proved its safety.

**TABLE 1:** Absorbed doses to target organs and effective dose.

Target organs	Organ doses (μGy/MBq)
Adrenals	17.0 ± 0.6
Brain	15.2 ± 1.0
Breasts	7.9 ± 0.3
Gallbladder wall	133.7 ± 60.9
Lower large intestine wall	11.8 ± 0.6
Small intestine	44.5 ± 5.4
Stomach wall	12.7 ± 0.2
Upper large intestine wall	18.9 ± 1.1
Heart wall	16.6 ± 0.7
Kidneys	31.6 ± 2.2
Liver	88.5 ± 11.2
Lungs	11.1 ± 0.2
Muscle	9.5 ± 0.3
Ovaries	13.7 ± 0.6
Pancreas	17.1 ± 0.6
Red marrow	11.3 ± 0.6
Osteogenic cells	11.7 ± 0.6
Skin	7.0 ± 0.3
Spleen	10.7 ± 0.3
Testes	7.7 ± 0.5
Thymus	9.2 ± 0.4
Thyroid	8.3 ± 0.5
Urinary bladder wall	10.6 ± 0.5
Uterus	13.2 ± 0.6
Parotid	14.3 ± 3.8
Total body	12.3 ± 0.1
Effective dose (mSv/MBq)	15.3 ± 0.3

### [^18^F]-S16 Visual Assessment

**FIGURE 2:**
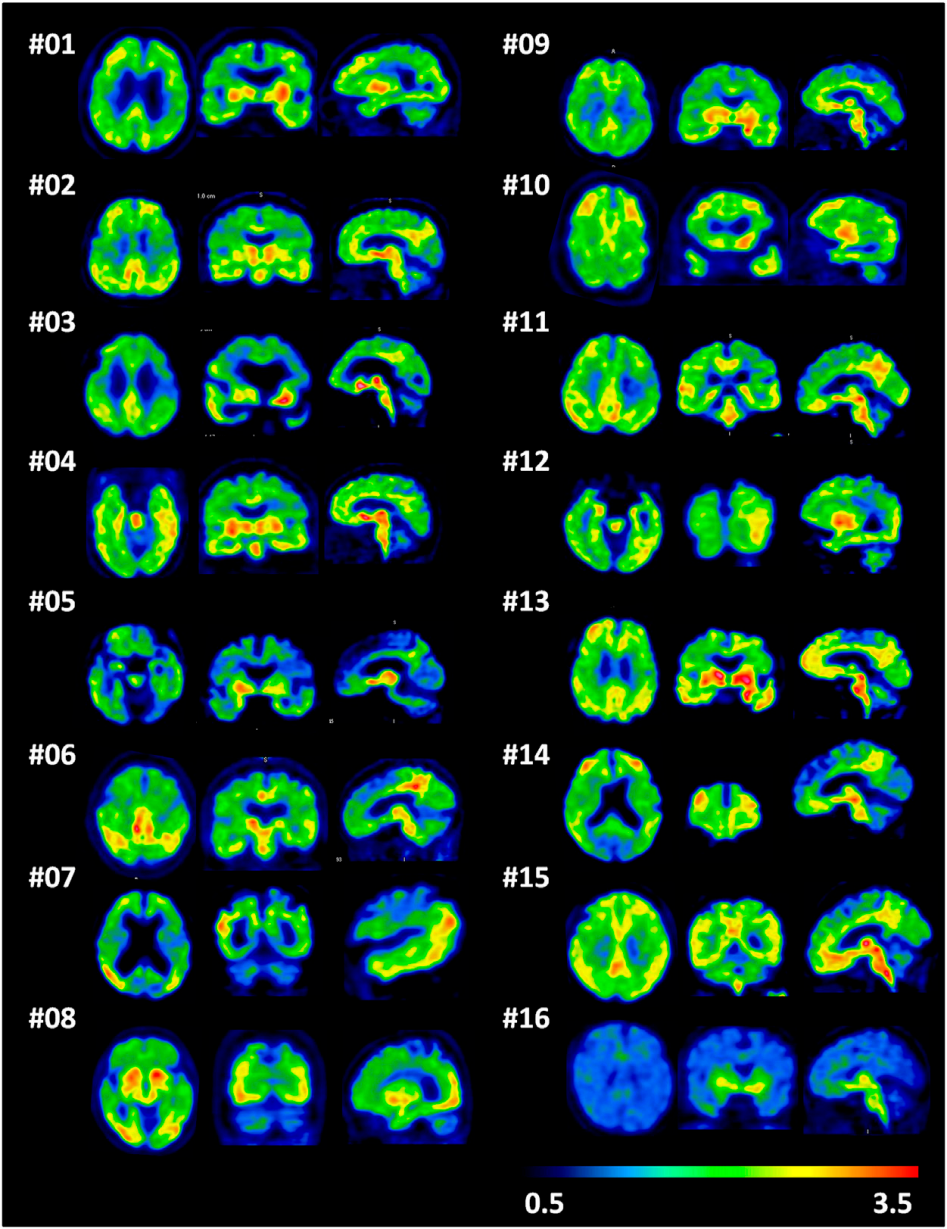
A single frame of [^18^F]-S16 positron emission tomography images (85–90 min) in a transverse, coronal, and sagittal plane for all Alzheimer’s disease patients (subjects 1–15) and one representative healthy control (subject 16).

The demographic and clinical characteristics of 15 AD subjects and 6 HCs are summarized in Supplementary Table S1. In HCs, no areas of specific [^18^F]-S16 cerebral uptake were identified visually (Figure 2), and a consistent pattern of initial uptake and washout was observed. In AD subjects, tracer accumulation was identified in cortical regions mainly involving the parietal and temporal lobes, precuneus, occipital lobes, and frontal lobes. One AD patient (subject #8) revealed some posterior cerebral artery (PCA) syndromes, showing a significantly higher [^18^F]-S16 retention in the occipital lobe. Two AD patients revealed notably higher S16 retention in the bilateral frontal lobe (subject #10) and lower glucose metabolism compared to other AD cases. In addition, subject #12 (male, age 64) displayed a higher S16 retention in the left posterior temporal and inferior parietal lobes as well as hypometabolism in the same regions. Radiotracer retention was observed in the substantia nigra and brainstem of both HCs and AD subjects. No binding higher than the uptake in the reference region was found in the choroid plexus, venous sinus, and white matter.

### ^18^F-S16 Binding Related to Clinical Diagnosis and Quantitative Analyses

**FIGURE 3:**
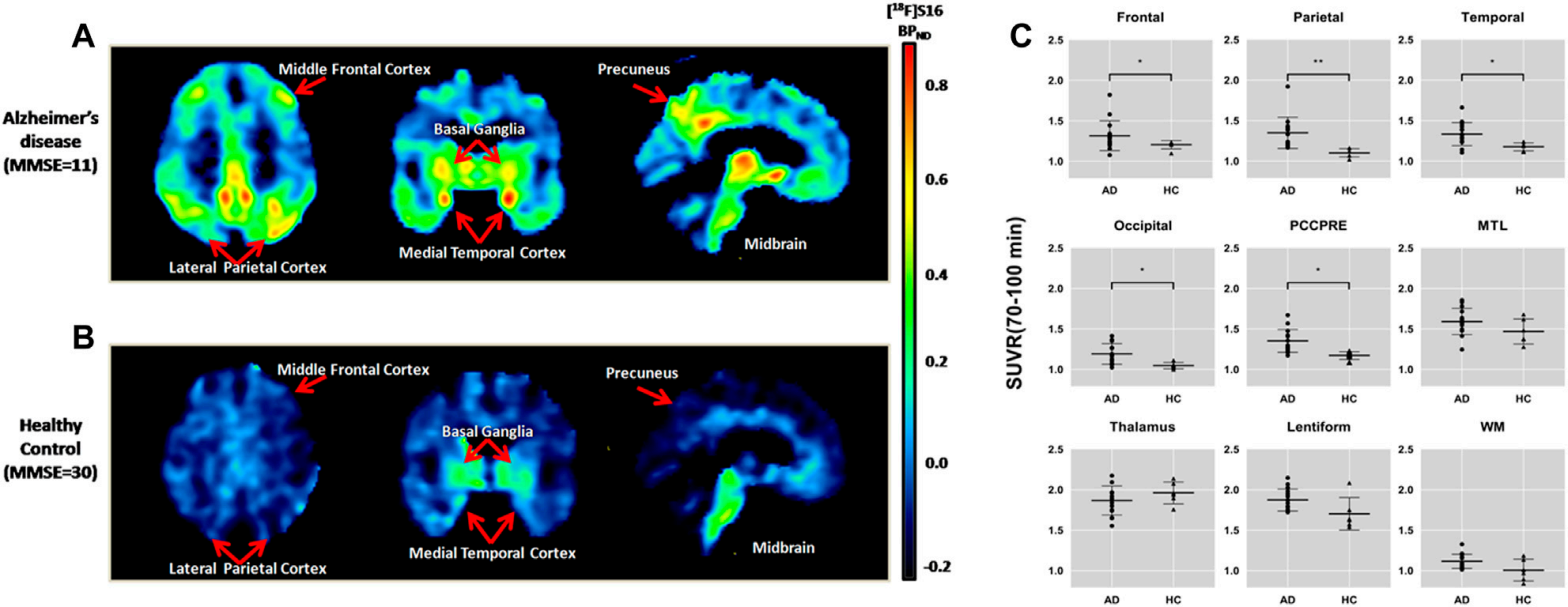
Comparison of an Alzheimer’s disease patient [subject 6, Mini-Mental State Examination (MMSE) = 11] **(A)** and a healthy control (subject 16, MMSE = 30) **(B)** in the mean [^18^F]-S16 BP_ND_ positron emission tomography maps. The images were normalized to the Montreal Neurologic Institute space and presented as a transverse, coronal, and sagittal plane. Box plot of standardized uptake value ratio (SUVR; 70–100 min) of each diagnosis group for different brain regions **(C)**. Box plot of SUVR (70–100 min) of each diagnosis group for different brain regions.*p < 0.05, **p < 0.01.

A comparison of a representative AD and HC subject in the mean [^18^F]-S16 BP_ND_ PET map is shown in Figures 3A,B. [^18^F]-S16 accumulation in AD subject #6 was identified in the cortical regions mainly involving the parietal, temporal, precuneus, and frontal lobes. No areas of specific [^18^F]-S16 cerebral uptake were identified visually in HC subject #16. [^18^F]-S16 retention was observed in the substantia nigra and brainstem of both AD and HC subjects.

The ROI analysis of SUVRs is summarized in Table 2. The SUVRs of AD patients ranged from 1.12 to 1.87 (white matter and lentiform, respectively). The lentiform, thalamus, and medial temporal lobe showed the greatest SUVRs in AD. Without the substantia nigra ROIs, the highest SUVR in AD was the medial temporal lobe (1.59), followed by posterior cingulate and precuneus (1.35), and parietal (1.35) and temporal lobe (1.33). The SUVRs for HC ranged from 1.01 (white matter) to 1.96 (thalamus). The lentiform, thalamus, and medial temporal lobe also presented with the highest SUVRs in HC. The region with the lowest SUVR in both AD and HC groups was the white matter. All ROIs had SUVRs that were greater than 1.0, indicating that the uptake of [^18^F]-S16 was greater than that of the cerebellar reference region.

**TABLE 2:** Standardized uptake value ratios.

ROI	AD	HCs
Frontal	1.32 (0.183)	1.21 (0.053)
Parietal	1.35 (0.192)	1.10 (0.050)
Temporal	1.33 (0.141)	1.18 (0.049)
Occipital	1.19 (0.127)	1.05 (0.039)
Posterior cingulate and precuneus	1.35 (0.141)	1.17 (0.048)
Medial temporal	1.59 (0.163)	1.47 (0.155)
Thalamus	1.87 (0.180)	1.96 (0.135)
Lentiform	1.88 (0.135)	1.70 (0.201)
White matter	1.12 (0.087)	1.01 (0.134)

Standardized uptake value ratios mean values (standard deviation) from 70 to 100 min using the cerebellar cortex as the reference region.

AD, Alzheimer’s disease; HCs, healthy controls; ROI, region of interest.

[^18^F]-S16 uptake in different ROIs of AD and HC subjects is shown as SUVR box plots in Figure 3C. The AD subjects showed generally higher SUVRs than HCs in the cortical regions, especially in the parietal lobe (*p* < 0.0001), posterior cingulate and precuneus (*p* < 0.001), lateral temporal and occipital lobes (*p* < 0.01), and frontal lobe (*p* < 0.05). However, there was no statistically significant difference in the medial temporal lobe. The SUVR analyses of the subcortical regions did not show statistically significant differences between AD and HC subjects in the thalamus and white matter. However, lentiform revealed a significant difference between AD and HCs (*p* < 0.05).

### [^18^F]-S16 Quantitative Analyses and Correlation With Glucose Metabolism

**FIGURE 4:**
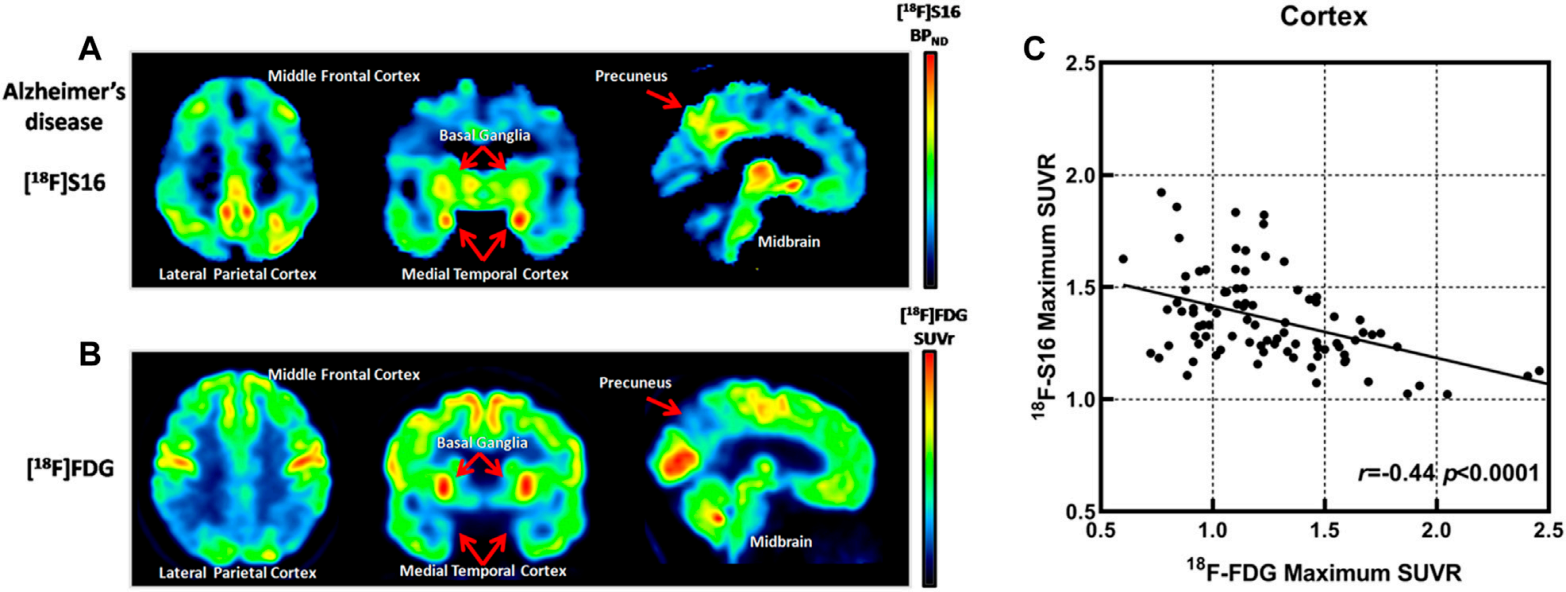
Comparison of [^18^F]-S16 BP_ND_ positron emission tomography (PET) map and [^18^F]-FDG SUVR PET map obtained in the same Alzheimer’s disease patient (subject 6). The images were normalized to the Montreal Neurologic Institute space and presented as a transverse, coronal, and sagittal plane **(A,B)**. Correlation plots for [^18^F]-S16 and [^18^F]-FDG PET. *r* = −0.44, *p* < 0.0001 **(C)**.

The SUVR values of the summed [^18^F]-S16 PET and [^18^F]-FDG PET image were related to all six cortical regions to investigate the regional relationship between tau deposition and glucose metabolism. Spearman rank-correlation coefficient (*r*) was compared between [^18^F]-S16 PET and [^18^F]-FDG PET for all cortical ROI regions in AD patients. The correlation coefficients were determined by comparing [^18^F]-S16 SUVR and [^18^F]-FDG SUVR (global mean and normalization). The correlation plots of [^18^F]-S16 PET and [^18^F]-FDG PET are shown in Figure 4. There was a significant difference in the correlation coefficients between [^18^F]-S16 and [^18^F]-FDG PET (*r* = −0.44, *p* < 0.0001) for global mean normalization.

## Discussion

In this study, we have reported the fully automated radiosynthesis and the first human PET study of [^18^F]-S16. No radioactive uptake in the bone of healthy volunteers guaranteed its stability *in vivo*. In addition, the suitable lipophilicity and molecular weight of [^18^F]-S16 resulted in their excellent characteristics of crossing the blood–brain barrier. The biodistribution of [^18^F]-S16 in humans was consistent with that of animal studies as previously reported. The line of activity seen in Figure 1A is tracer accumulation in the vein, which is obvious soon after injection but gradually disappears. The bladder did not show intensive uptake throughout the study. As a result, we deduced that [^18^F]-S16 was mainly excreted through the hepatic–biliary system, resulting in a high accumulation in the liver and gall bladder. The radioactive uptake in the gall bladder, colon, and small intestine increased rapidly, while that in the kidneys and bladder decreased sharply. The time–distribution pattern of [^18^F]-S16 in the gall bladder is shown in Figure 1B (marked with red arrows). The uptake in the gall bladder was low at 10 min post-injection, and it increased to a high level at 60 and 120 min when the probe was gradually secreted into the gall bladder cavity. At 240 min post-injection, the probe was almost fully secreted into the gall bladder.

The effective dose of [^18^F]-S16 was 15.3 ± 0.3 μSv/MBq, which is in the typical range for F-18 radioligand (Zanotti-Fregonara et al., 2013). The dosimetry data of [^18^F]-S16 showed a slightly lower effective dose compared to that of other tau tracers (22.5 ± 3.6 μSv/MBq for [^18^F]-AV1451, 22.7 ± 1.3 μSv/MBq for [^18^F]-THK5351, and 29.4 ± 0.6 μSv/MBq for [^18^F]-MK6240) (Choi et al., 2016; Hsiao et al., 2017; Koole et al., 2020).

The present study showed the accumulation of [^18^F]-S16 in regions known to have tau deposition in Aβ-positive clinically probable AD subjects. The HC subjects showed a very low [^18^F]-S16 accumulation in all cortical brain regions, and the AD subjects could be clearly distinguished. In the AD group, all cortical ROIs revealed average SUVR values over 1.19, especially in significant brain areas of the medial temporal lobe, posterior cingulate and precuneus, parietal lobe, lateral temporal, frontal lobe, and occipital lobes associated with tau deposition (Braak and Braak, 1997). The highest uptake of [^18^F]-S16 was observed in the medial temporal lobe (including entorhinal cortex, hippocampus, and parahippocampus). A higher uptake of [^18^F]-S16 was observed in the posterior cingulate and precuneus, lateral temporal, and parietal lobe. The spatial distribution observed here parallels neuropathologic data reporting tau spread from the entorhinal cortex through the inferior-lateral temporal and medial parietal areas to the neocortex (Braak et al., 2006). The distribution and density of tracer uptake in AD subjects observed in this study are also consistent with previous tau PET imaging studies (Jack et al., 2018; Ossenkoppele et al., 2018). Besides this, the present study revealed that tracer binding predominantly occurred in the gray matter of the AD group. The white matter showed the lowest uptake of [^18^F]-S16 both in the AD and HC groups. High gray-to-white-matter ratios in the AD cases reflect radiotracer binding to the cortical neurons that are most affected and are in good agreement with the excellent contrast observed in human PET scans.

The uptake of [^18^F]-S16 was significant in the MTL of HCs, which was consistent with previously reported tau tracers. Tau imaging in cognitively normal elderly individuals using [^18^F]-THK series and [^18^F]-AV1451 showed radioactivity retention in the MTL (Chiotis et al., 2016; Cho et al., 2016). This pattern of MTL binding is consistent with the neuropathological literature (Braak et al., 2011) and may reflect an age-related process of tau accumulation in this region (Tomlinson et al., 1970), the so-called primary age-related tauopathy (Jellinger et al., 2015), which has been shown to result in hippocampal atrophy and mild amnestic deficits that are independent of amyloid-β (Josephs et al., 2017). The age-related accumulation of tau, followed by the spreading of tau potentiated by Aβ, disrupts functional memory circuits by disconnecting key MTL structures. Tau pathology plays a role in disconnecting the hippocampus from specific MTL memory systems, leading to increased local coherence and memory decline (Harrison et al., 2019).

In this study, we found that one AD patient showed a significantly higher tracer retention in the occipital lobe (subject #8). This patient revealed PCA syndromes. PCA is a focal neurodegenerative disorder of higher visual processing and spatial praxis with relative sparing of memory and insight, which is considered one subtype of variant AD. In PCA, a high consistency between the phospho-tau-specific antibody AT8 immunohistochemical staining and tau tracer binding was observed (Yap et al., 2021). Tau-PET imaging showed significantly higher binding compared to patients with similar clinical features arising from dementia with Lewy bodies (Nedelska et al., 2019). Patients with PCA presented with a higher uptake in the occipital and parietal brain regions, while patients with amnestic-predominant presentation showed the highest binding in the medial temporal and lateral temporoparietal regions (Ossenkoppele et al., 2016). Our data was consistent with previous immunohistochemical and tau-PET studies. We found that two AD patients revealed notably higher S16 retention in the bilateral frontal lobe (subject #10) and lower glucose metabolism compared to other AD cases. The clinical presentations of these two patients showed dysexecutive deficits and impulsive and repetitive movements. They may belong to the behavioral variant of Alzheimer’s disease, which represents another rare variant of AD. It was characterized by early and predominant behavioral deficits and personality changes caused by the AD pathology. In addition, subject #12 (male, age 64) presented with language impairment in fluency and sentence repetition. He displayed higher S16 retention in the left posterior temporal and inferior parietal lobe as well as hypometabolism in the same regions. This patient may belong to the language variant of Alzheimer’s disease.

We observed the strongest binding for the lentiform and thalamus both in the AD and HC groups, which might be largely associated with off-target binding. Off-target binding is a major limitation associated with the first-generation tau radioligands. In particular, the basal ganglia and thalamus were found to be affected by extensive binding to monoamine oxidase B (MAO-B) (Yap et al., 2021). MAO-B is a flavoenzyme in the mitochondrial outer membrane and plays a major role in the metabolism of neuroactive and vasoactive amines. In previous studies, MAO-B binding was also considered as an important off-target binding of THK probes. A previous study from Prof. Cui indicated that the [^18^F]-S16 shared the same THK binding site of NFTs (Zhou et al., 2021). We followed the successful optimization strategy of THK derivatives in order to achieve an appropriate tau-targeting probe with high binding affinity and selectivity over Aβ plaques and with low off-target binding and suitable *in vivo* pharmacokinetics. When a unique fluoropropanol side chain was introduced to the THK scaffold, to give [^18^F]THK-5117 and [^18^F]THK-5351, they showed higher selectivity for tau and more favorable *in vivo* kinetics. We introduced the chiral fluoropropanol side chain at the phenolate position and N,N-dimethylamino/N-monomethylamino group at position 6 or 7 of the 2-phenylquinoxaline scaffold to find compounds with increased binding affinity and selectivity toward NFTs. Thus, [^18^F]-S16, which was a [^18^F]-labeled chiral 2-phenylquinoxaline derivative, was designed, synthesized, and evaluated as a PET imaging probe for tangles. We found that this quinoxaline derivative was capable of binding with NFTs on the brain sections of AD patients *in vitro*. The parallel comparison between [^18^F]-S16 and [^18^F]-THK5351 revealed that S16 had many advantages, such as high binding affinity, selectivity, initial brain uptake, and low off-target binding (Zhou et al., 2021). As such, there was lower [^18^F]-S16 binding in the MAO-B-rich lentiform in HCs than in the AD group in this study.

In this study, we directly compared [^18^F]-S16 SUVR and [^18^F]-FDG SUVR for all cortical ROIs in AD patients. Studies have shown a significant correlation between [^18^F]-S16 binding and hypometabolism across neocortical regions. This finding was consistent with other tau radioligand PET studies (Ossenkoppele et al., 2015; Bischof et al., 2016; Ossenkoppele et al., 2016; Dronse et al., 2017; Whitwell et al., 2018). The A/T/N classification scheme suggests that the tau pathology follows in the time course of disease development after amyloid-β pathology. Early Aβ deposition occurs across much of the cortex even in clinically normal aging (*e*.*g*., preclinical AD), while tau pathology begins in the transentorhinal cortex in the MTL and may spread in an activity-dependent manner along vulnerable functional networks (Harrison et al., 2019). Animal studies have indeed shown the spread of tau pathology *via* synaptic connections, with this process accompanied by neurodegenerative changes (de Calignon et al., 2012; Ahmed et al., 2014). The tau protein plays a key role in the formation of intraneuronal NFTs and might represent an important therapeutic target in AD because tau deposition is highly associated with neurodegeneration and cognitive decline. Our findings clearly demonstrate that [^18^F]-S16 PET can provide information on “T”. [^18^F]-S16 tau PET imaging has the potential to facilitate an accurate diagnosis of tauopathy, a precise assessment of disease severity and disease progression, and a specific appraisal of efficacy of potential disease-modifying anti-tau treatments.

## Conclusion

[^18^F]-S16 could be automatically synthesized on a large scale, and it shows favorable biodistribution and safety to the human body. The dosimetry data are comparable to that of other tau tracers. The AD subjects show a generally higher [^18^F]-S16 uptake than HCs in the cortical regions, and a significant correlation between [^18^F]-S16 binding and hypometabolism across neocortical regions could be observed. We find prominent [^18^F]-S16 uptake in regions where abnormal tau aggregates are expected to accumulate. Our results suggest that the new tau PET tracer is a suitable tool to distinguish AD and HCs. It is necessary to conduct more studies on more subjects and involve other tauopathy reactions.

## Data Availability

The original contributions presented in the study are included in the article/Supplementary Material. Further inquiries can be directed to the corresponding author.
