# Supplementary material for: Biodistribution and Dosimetry Evaluation for a Novel Tau Tracer [18F]-S16 in Healthy Volunteers and Its Application in Assessment of Tau Pathology in Alzheimer’s Disease
Source: Front Bioeng Biotechnol. 2022 Feb 10;9:812818. doi: 10.3389/fbioe.2021.812818 (PMC8866701; doi:10.3389/fbioe.2021.812818)
Supplement: Supplementary file 1 [file DataSheet1.doc]

Supplementary Materials

**Biodistribution and Dosimetry Evaluation for A Novel Tau Tracer [^18^F]-S16 in Healthy Volunteers and Its Application in Assessment of Tau Pathology in Alzheimer’s Disease**

Ying Wang^1^, Li Cai^1^, Kaixiang Zhou^2^, Mengchao Cui^2^, Shaobo Yao^3*^

^1^ Department of PET/CT Diagnostic, Tianjin Medical University General Hospital, Tianjin, 300052, China

^2^ Key Laboratory of Radiopharmaceuticals, Ministry of Education, Beijing Normal University, Beijing, 100875, China

^3^ Department of Nuclear Medicine, Fujian Provincial Key Laboratory of Precision Medicine for Cancer, The First Affiliated Hospital of Fujian Medical University, Fuzhou, Fujian, 350005, China


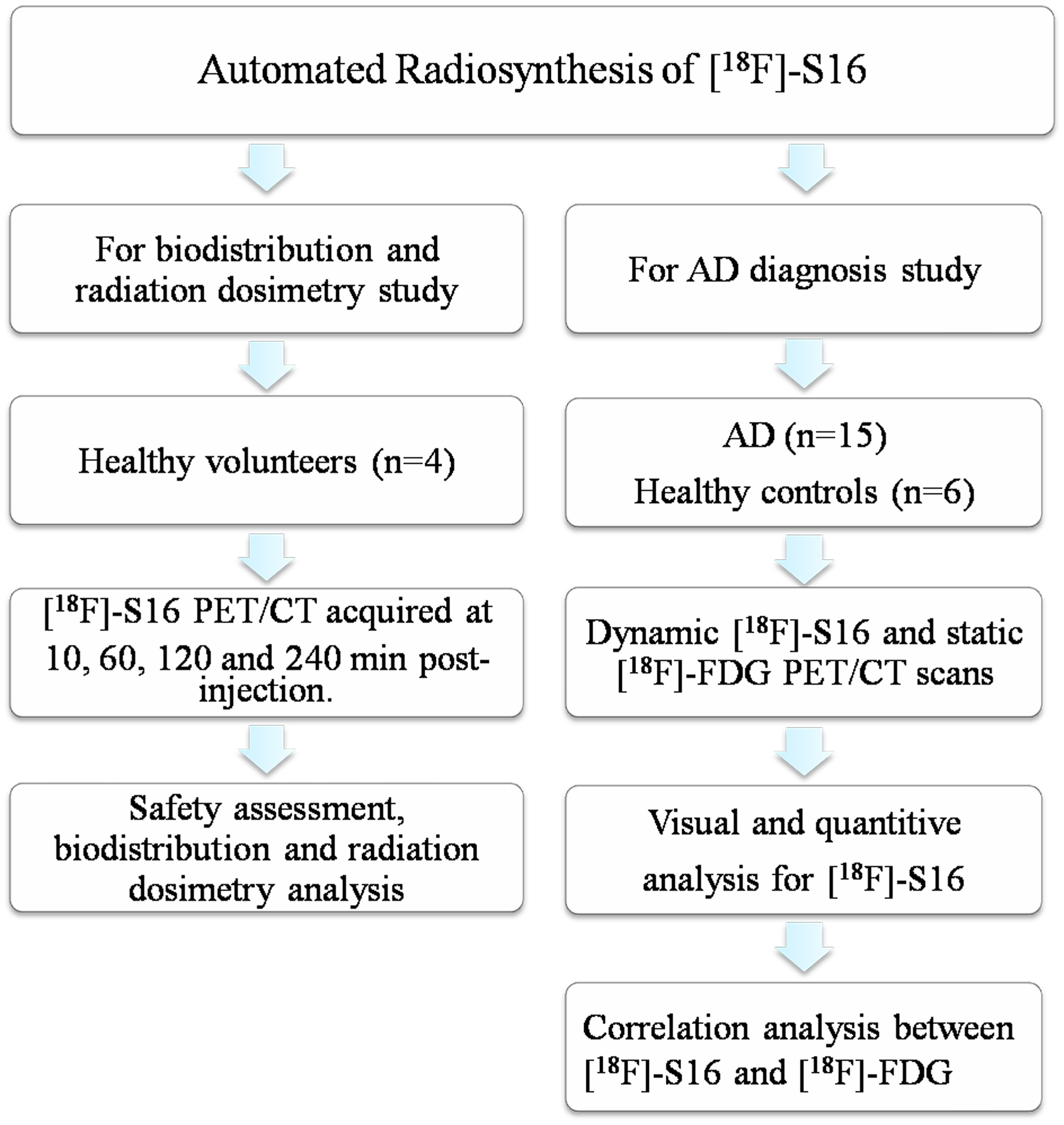


**Fig. S1** The flowchart of study design. The automated radiosynthsis of [^18^F]-S16 was performed. And then four healthy volunteers were underwent a series PET scans acquired at 10, 60, 120 and 240 min post-injection. The biodistribution in normal organs and safety were assessed. Radiation dosimetry was calculated. For AD diagnosis study, fifteen AD and six HCs underwent dynamic [^18^F]-S16 and static [^18^F]-FDG PET imaging. [^18^F]-S16 binding was assessed visually and quantitatively. Spearman rank correlation analysis was used to calculate the voxel-wise correlations between [^18^F]-S16 PET and [^18^F]-FDG.


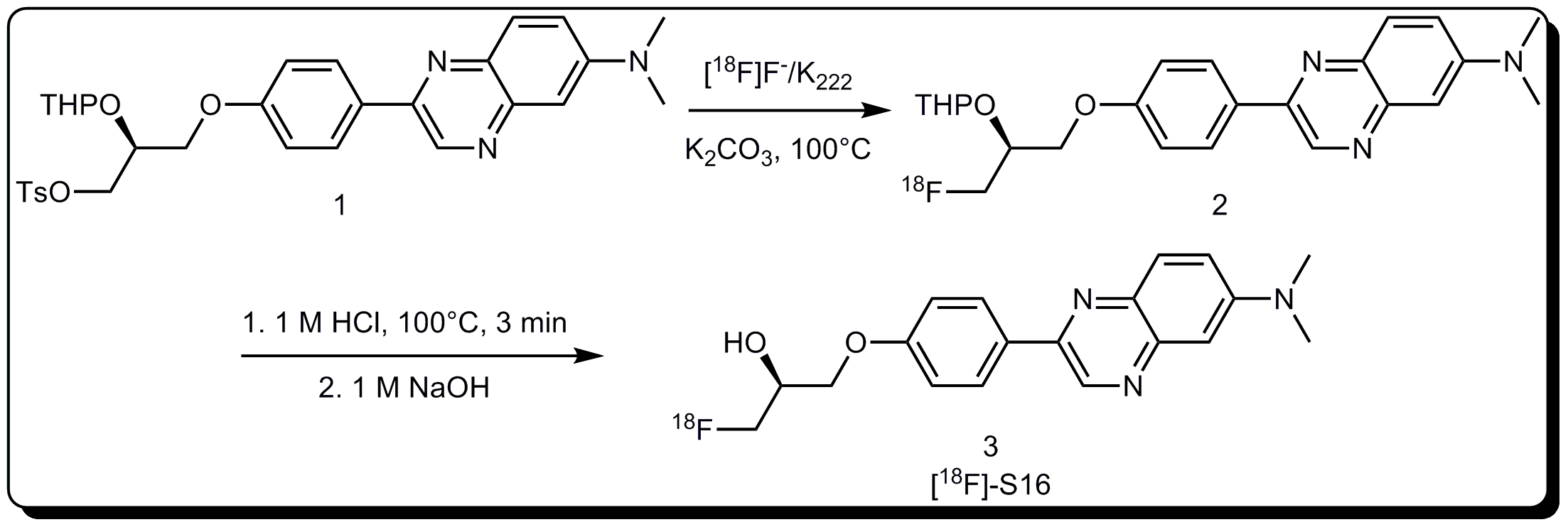


**Fig. S2** Schematic diagram of the radiosynthesis of [^18^F]-S16. The reaction conditions for preparation of [^18^F]-S16 was discussed. The phase-transfer-catalyst, precursor amount, reaction solvent and time were confirmed to be K_222_/K_2_CO_3_, 3 mg, CH3CN and 5 min.


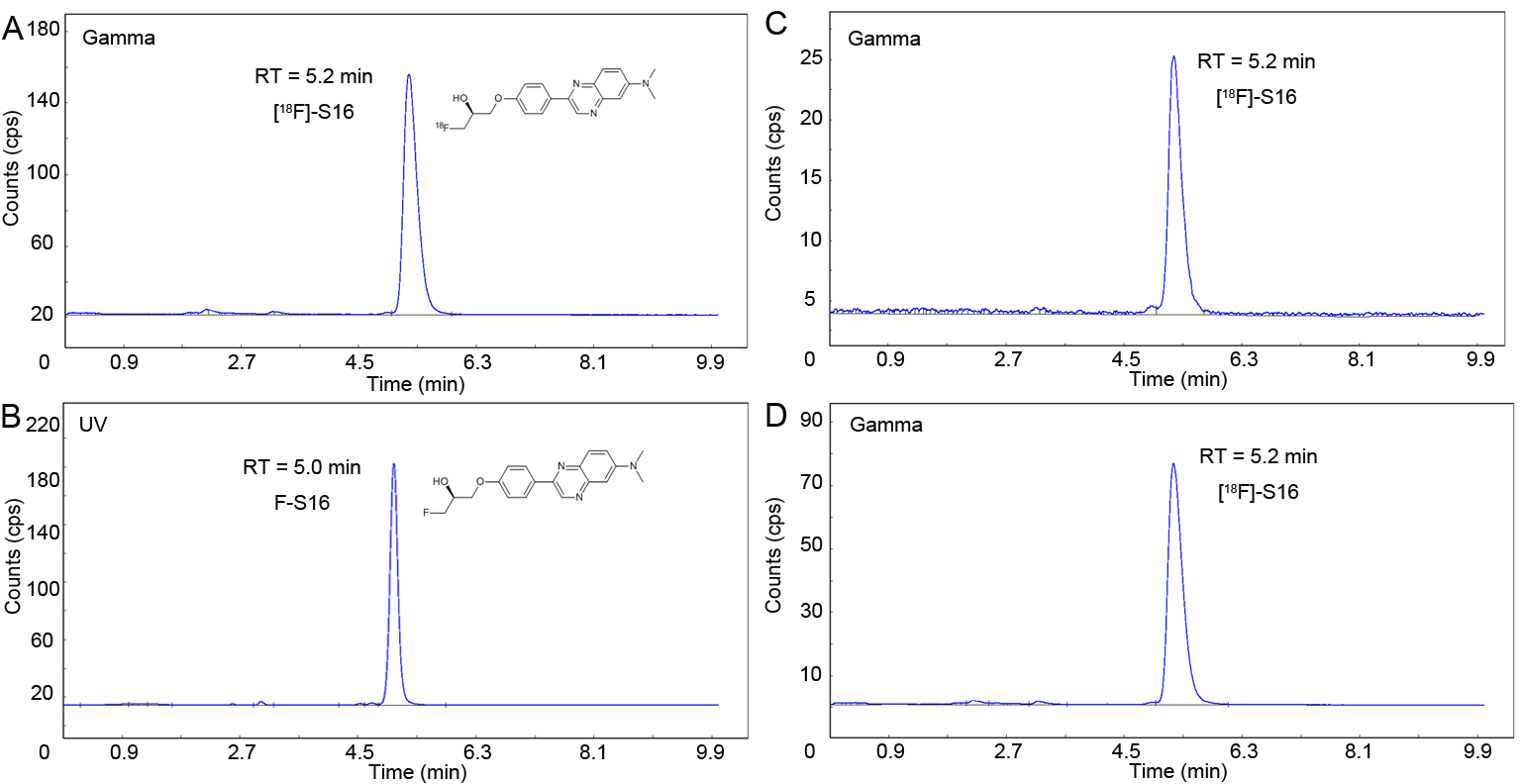


**Fig. S3** Gamma (**A**) and UV (**B**) HPLC chromatograms of the purified [^18^F]-S16 injection saline co-injected with the cold standard [^19^F]-S16. Radio-HPLC evaluation of the in vitro stability of [^18^F]-S16 incubated at 37ºC in FBS (**C**) and in saline (**D**) for 120 min.


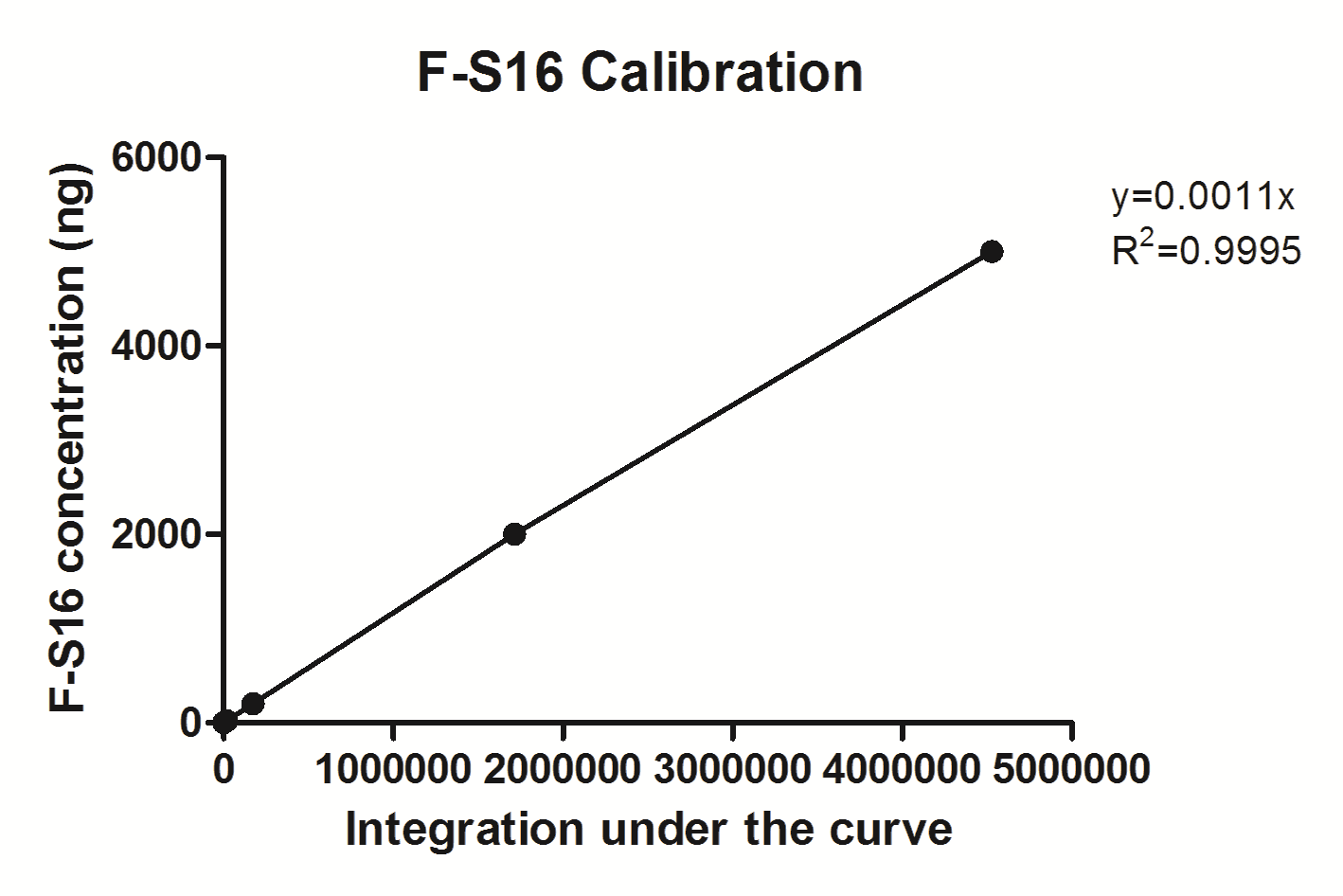


**Fig. S4** With an integration of 5157 (area under the curve), the concentratino of [^18^F]-S16 (refered to as F-S16 in the graph) 5.7 ng. The overall specific activity was determined to be 1480 GBq/μmol. The average specific activity 1047 ± 450 GBq/μmol for synthesis ranging from 30-37 GBq of n.c.a [^18^F]-Fluoride as starting activity

**Table S1.** Demographic and clinical characteristics of participants

| **Subject**  **NO.** | **Gender** | **Age(y)** | **Cohort** | **Injected**  **dose**  **(MBq)** | **Amyloid**  **Status**  **(visual)** | **MMSE**  **Score** | **CDR**  **Score** |
| --- | --- | --- | --- | --- | --- | --- | --- |
| **1** | Male | 68 | AD | 399.6 | Positive | 21 | 1 |
| **2** | Male | 54 | AD | 384.8 | Positive | 18 | 1 |
| **3** | Female | 71 | AD | 362.6 | Positive | 20 | 0.5 |
| **4** | Male | 77 | AD | 329.3 | Positive | 19 | 0.5 |
| **5** | Female | 54 | AD | 373.7 | Positive | 21 | 0.5 |
| **6** | Female | 55 | AD | 340.4 | Positive | 11 | 2 |
| **7** | Female | 64 | AD | 414.4 | Positive | 15 | 1 |
| **8** | Female | 60 | AD | 418.1 | Positive | 19 | 1 |
| **9** | Male | 68 | AD | 377.4 | Positive | 26 | 0.5 |
| **10** | Male | 57 | AD | 444.0 | Positive | 10 | 2 |
| **11** | Female | 41 | AD | 384.8 | Positive | 25 | 0.5 |
| **12** | Male | 64 | AD | 395.9 | Positive | 11 | 1.5 |
| **13** | Female | 79 | AD | 444.0 | Positive | 23 | 0.5 |
| **14** | Female | 57 | AD | 370.0 | Positive | 14 | 1 |
| **15** | Female | 66 | AD | 329.3 | Positive | 27 | 0.5 |
| **16** | Female | 56 | HC | 388.5 | Negative | 30 | 0 |
| **17** | Female | 61 | HC | 373.7 | Negative | 30 | 0 |
| **18** | Male | 63 | HC | 381.1 | Negative | 30 | 0 |
| **19** | Female | 72 | HC | 381.1 | Negative | 29 | 0 |
| **20** | Female | 60 | HC | 370.0 | Negative | 30 | 0 |
| **21** | Male | 55 | HC | 392.2 | Negative | 30 | 0 |

In total, 15 AD subjects and 6 HCs were included in this analysis. Subject demographics and clinical characteristics are presented in Table 1. Mean age was 62.3 ± 9.9 for AD subjects and was 61.2 ± 6.1 for HCs. There were no significant differences in age and gender (*p*>0.05). Mean MMSE scores in the AD and HCs groups were18.6 (range, 10-27) and 29.8 (range, 29-30). Mean CDR scores in the AD and HCs groups were1.0 (range, 0.5-2.0) and 0. MMSE score and CDR score in the HCs group were higher than AD group (*p*<0.05). The amyloid status were positive in AD group and were negative in HC group by visual assignment.)
